# Emerging Role of MicroRNA-200 Family in Dentistry

**DOI:** 10.3390/ncrna7020035

**Published:** 2021-06-11

**Authors:** Pei-Ling Hsieh, Chun-Chung Huang, Cheng-Chia Yu

**Affiliations:** 1 Department of Anatomy, School of Medicine, China Medical University, Taichung 404333, Taiwan; plhsieh@mail.cmu.edu.tw; 2 Institute of Oral Sciences, Chung Shan Medical University, Taichung 40201, Taiwan; seanabo@gmail.com; 3 Department of Dentistry, Chung Shan Medical University Hospital, Taichung 40201, Taiwan; 4 School of Dentistry, Chung Shan Medical University, Taichung 40201, Taiwan

**Keywords:** miR-200 family, oral cancer, oral potentially malignant disorders

## Abstract

MicroRNAs (miRNAs) are endogenous non-coding RNAs ~22 nucleotides in length, which have been shown to participate in various biological processes. As one of the most researched miRNAs, the miR-200 family has been found to regulate several factors that are associated with the epithelial to mesenchymal transition (EMT) and cancer stem cells (CSCs) behavior. In this review, we briefly summarize the background of the miR-200 family and their implication in various dental diseases. We focus on the expression changes, biological functions, and clinical significance of the miR-200 family in oral cancer; periodontitis; oral potentially malignant disorder; gingival overgrowth; and other periodontal diseases. Additionally, we discuss the use of the miR-200 family as molecular biomarkers for diagnosis, prognostic, and therapeutic application.

## 1. Introduction

Previous studies classified most RNAs as mRNA, rRNA, and tRNA. Messenger RNA (mRNA) transcripts gene messages, and ribosomal RNA (rRNA) is self-catalytic and provides ribosomal structures [1]. Transfer RNA (tRNA) translates mRNA into protein synthesis. Other RNAs also participate in modification and message transmission [2,3] and many non-coding protein RNA genes in the genomes of animals and plants produce RNA with a length of approximately 19–25 nucleotides. This small RNA regulates the expression of other genes and plays an important role in the development of organisms.

In 1993, Ambros et al. discovered the first miRNA molecule: lin-4 in *C. elegans* [4]. There was no significant progress in the study of microRNA until 2000, when Ruvkun et al. discovered the microRNA molecule 1et-7 [5]. MicroRNA has become the subject of many studies. After a few decades of rapid advances, research into non-coding RNAs (ncRNAs), which are non-coded small RNA molecules that regulate gene expression, has generated a rich and complex body of knowledge and has shown that non-coding RNAs play a key role in many diseases that involve dynamic changes in the genome [6,7,8].

Non-coding RNAs that produce oncogenes with dominant gain-of-function mutations sometimes act as tumor suppressors, depending on the main target genes [9]. Both classes of functions have been identified through their alteration in human and animal cancer cells in experimental models. Studies showed that microRNAs are closely related to physiological phenomena, such as development, hematopoiesis, organ formation, apoptosis proliferation, and tumor growth, and can be used in the diagnosis and treatment of diseases [10]. Other studies showed that the aberrant expression of these ncRNAs can act as a surrogate diagnostic marker or as a therapeutic target for cancer treatment.

In this study, we reviewed previous studies by us and others to determine how these ncRNAs affect oral cancer stemness and their potential clinic applications [11,12,13]. A better understanding of the implication of these ncRNAs in oral tumor genesis allows basic ncRNA research to be translated into clinical applications. Non-coding RNAs are RNA that does not activate for protein synthesis. These are classified as small non-coding RNAs (such as microRNAs, ~19–22 nucleotides in length) and long non-coding RNAs (lncRNAs, more than 200 nucleotides in length) [14].

MicroRNAs (miRs) regulate the translational efficiency or stability of targeted mRNAs by interacting with the targets of the 3′ untranslated region (3′-UTR) [15]. There are more than 60,000 long non-coding RNAs (lncRNAs) and they are the most abundant ncRNA families in humans [16,17]. Compelling evidence shows that lncRNAs play a critical role in regulating gene expression, mainly through *cis*-regulation or *trans*-regulation [18]. lncRNAs are implicated in various levels of fundamental epigenetic processes in a different way from which miRs post-transcriptionally regulate mRNAs [19].

lncRNAs can act as molecular signals or as decoys for miR target sites. lncRNAs can also function as a guide to recruit chromatin-modifying enzymes to target genes or as scaffolds to form ribonucleoprotein complexes. Transcriptomic sequencing using next-generation sequencing (NGS) shows that only a small proportion of the identified lncRNAs in humans may be biologically relevant [20]. Although their function has been reported at all levels of gene regulation, including epigenetic, transcriptional, translational, and post-translational actions, the underlying mechanisms through which lncRNA affects gene regulation is still largely unknown [18,21].

It has recently been shown that lncRNAs are involved in the onset and pathophysiology of different types of oral diseases. Deregulated lncRNA expression patterns have been proven to significantly interfere with cancer hallmarks, including aberrant tumor cell proliferation and tumor aggressiveness, which increase the risk of metastasis [22,23]. As one of the most researched miRNAs, the miR-200 family has been found to regulate several factors that are associated with the epithelial-to-mesenchymal transition (EMT) and cancer stem cells (CSCs) behavior.

The miR-200 family is composed of two gene clusters, miR-200a/miR-200b/miR-429 located on human chromosome 1p36.33 and miR-200c/miR-141 on chromosome 12p13.31. The miR-200 family was recently revealed to regulate various genes that are responsible for the different dental epithelial cell lineages [24]. It was proven that miR-200s modulate Sox2 expression in the stem cell niche and affect the incisor tissue composition and enamel formation [24]. miR-200s not only play important roles in epithelial cell differentiation and tooth development [25], they are also involved in various pathological conditions in the oral cavity, such as periodontitis [26] and oral cancer [9].

Accumulating evidence suggests that miR-200 family members participate in the decision of the epithelial phenotype of cancer cells as well as their metastasis capacity [27,28]. One of the possible mechanisms is that the miR-200 family may regulate the process of EMT, and it is well-known that the E-cadherin repressors, Zinc finger E-box-Binding homeobox 1 (ZEB1) and ZEB2, are both the targets of miR-200s [29,30]. Moreover, it was demonstrated that miR-200s also affect the homeostasis of the microenvironment via the regulation of protein shedding and secretion in lung cancer cells [31]. The miR-200 family was revealed to respond to chemo/radiotherapy following the accumulation of oxidative stress, which may contribute to drug resistance [32].

In this review, we briefly summarize and discuss the effects of the miR-200 family in various dental diseases and highlight their role in oral squamous cell carcinoma (OSCC). We aimed to provide valuable insight into the significance of miR-200s in dentistry. The upregulated and downregulated miR-200s are listed in Table 1.

## 2. microRNA-200 (miR-200) family

### 2.1. Expression Levels of the miR-200 Family

The different expression signatures of miR-200s are reported in various types of cancer. It was shown that miR-200s are aberrantly upregulated in ovarian and [66] breast [67] cancers, whereas the expression of miR-200s is downregulated in gastric [68] and hepatocellular [69] cancers. In OSCC, it was shown that the expression of miR-200b was inhibited in around 60% of the tumor samples [70]. Jensen et al. revealed that the expressions of miR-200c and miR-141 were downregulated at the invasive front of OSCC specimens, concomitant with the upregulated expression of EMT transcription factors ZEB1 and PRRX1 (Paired Related Homeobox 1) [49]. Arunkumar et al. showed that miR-200a/miR-200b/miR-429 and miR-141/miR-200c were all downregulated in OSCC tissues, and the reduction in the miR-200 family was associated with the risk habits of tobacco chewing and smoking [47].

Chronic exposure to chewing tobacco/cigarette smoke led to the dysregulated expression of miR-200a-3p, miR-200b-3p, miR-200c-3p, and miR-141-3p in oral keratinocytes [48]. Aside from behavioral risk factors, the miR-200 family is also regulated by various factors, such as grainyhead-like 2 (GRHL2), through direct promoter DNA binding in OSCC [71]. Conversely, infection with *Porphyromonas gingivalis* is also associated with the development of OSCC. It was demonstrated that *P. gingivalis* initiates the changes in EMT through upregulating ZEB1 promoter activity, which in turn reduces the levels of miR-200b, miR-200c, and miR-205 in gingival epithelial cells [35].

### 2.2. Biological Functions of the miR-200 Family

#### 2.2.1. miR-200 Family, EMT, and Cancer Stemness

It is well-known that the miR-200 family suppresses the EMT by inhibiting E-cadherin transcriptional repressors ZEB1 and ZEB2 in various types of cancers [9,28]. In OSCC, overexpression of miR200c/miR-141 was shown to impair the cell migration of tongue squamous cell carcinoma (UT-SCC-24A and 24B) cells, but not alter the proliferation and invasion abilities. Enforced expression of miR-200c diminished the expression of ZEB1 (not ZEB2); however, the regulation of miR-200c/miR-141 was blocked by hypermethylation of ZEB1 and ZEB2 [34]. Apart from EMT regulators, the miR-200 family may also modulate various pathways of cancer stemness, such as Notch or Oct4/Sox2 signaling [52,72]. In chemotherapy-resistant tongue squamous cell carcinoma CAL27 cells, miR-200b and miR-15b were the most significantly downregulated microRNAs [36]. Sun et al. demonstrated that suppression of miR-200b and miR-15b in the sensitive lines induced EMT and conferred chemoresistance, which may be through targeting B lymphoma Mo-MLV insertion region 1 homolog (BMI1) [36].

The oncogenic role of BMI in oral carcinogenesis has been reported in various studies as it may enhance cancer stemness [73,74], and BMI1-positive cells in the lingual epithelium were suggested to be the cancer stem cells (CSCs) in tongue cancer [75]. The elevated expression of BMI1 and decreased expression of miR-200c were observed in CSC-like ALDH1+/CD44+ cells [46]. Lo et al. revealed that miR-200c directly targeted the 3’ UTRs of BMI1, which affected the in vitro tumorigenic properties, self-renewal, metastasis, and chemo/radioresistance. Additionally, they demonstrated that overexpression of miR-200c repressed the expressions of ZEB1, Snail, and N-cadherin in CSC-like cells [46].

In addition to ZEBs and BMI1, miR-200b was indicated to directly bind to the 3’ UTRs of both ZEB2 and Kindlin-2 in tongue squamous cell carcinoma Tca-8113 cells [37]. Kindlin-2 is involved in the invasion-metastasis cascade of cancer progression via several pathways, such as TGFβ, Wnt/β-Catenin, Hedgehog, p53, and so on (see the review in [76]). A newly identified tumor suppressor, QKI, was also found to be regulated by miR-200. It was shown that knockdown of QKI elicited EMT and induced cancer cell invasion of CAL27 and HSC3 cells. Furthermore, they revealed a negative feedback loop between EMT/miR-200 and QKI in OSCC [50]. One of the recent studies using high-throughput screening to explore the potential genes involved in cisplatin resistance of OSCC revealed that Notch1, Jun, and ETS1 were the hub genes in the occurrence of cisplatin resistance, and these genes may be regulated by the members of the miR-200 family [51]. Further investigation is required to elucidate the relationship between miR-200s and these downstream factors as well as their effects on oral carcinogenesis.

#### 2.2.2. miR-141 and Epidermal Growth Factor Receptor (EGFR)

It was revealed that EGFR is a direct target of miR-141 [33], and EGFR was found to enhance tumor growth, invasion, and metastasis [77]. Zhao et al. reported that overexpression of miR-141 reduced the EGFR expression in OSCC cells [33]. They suggested that miR-141 impeded cell proliferation by downregulating cyclin-dependent kinase (CDK)-4 and induced apoptosis by repressing BCL-2. They indicated that miR-141 inhibited cell migration and invasion via suppressing matrix metalloproteinase (MMP)-2 [33]. Given that CDK4, BCL-2, and MMP2 were all modulated by EGFR, they extrapolated that the inhibitory effect of miR-141 on OSCC may be due to EGFR.

#### 2.2.3. miR-200 Family and lncRNAs

Emerging evidence has suggested that miR-200s may interact with various long non-coding RNAs (lncRNAs, >200 nucleotides) in different types of cancers. For instance, the oncogenic lncRNA OIP5-AS1 was shown to increase the expression of fibronectin by sponging miR-200b-3p, resulting in doxorubicin resistance in osteosarcoma cells [39]. As lncRNA OIP5-AS1 was upregulated in undifferentiated oral tumors [38], it is reasonable to presume that the regulatory effect of miR-200b may be suppressed in OSCC. Several studies revealed that miR-200c interacted with lncRNA MALAT-1 and formed a feedback loop in ovarian cancer [78] or pancreatic ductal adenocarcinoma [42]. It was shown that MALAT-1 inhibited the expression of miR-200c-3p, leading to the upregulation of ZEB1 expression. Likewise, overexpression of miR-200c-3p repressed MALAT-1 expression [42]. Numerous studies suggested that MALAT-1 may contribute to the development and progression of OSCC through the regulation of various axes, such as miR-125b/STAT3 [40] or miR-101/EZH2 [41]. The interplay between miR-200c and MALAT1 in OSCC is worthwhile of investigation.

### 2.3. Clinical Significance of the miR-200 Family

Arunkumar et al. showed that miR-200a and miR-200c are two markedly deregulated members of the family and related to the undifferentiated pathology. However, they observed no significant associations between the expression levels of the miR-200 family and tumor grade as well as the nodal stage in OSCC [47]. Another study reported the opposite results, and indicated that lower expression of miR-200b-5p in OSCC tissues was related to higher tumor grade [43]. Downregulation of miR-200c in OSCC tissues also correlated with advanced TNM stage and poor differentiation grade [45]. Furthermore, a meta-analysis suggested that the decreased expression in miR-200c was associated with poor prognosis [44].

## 3. Periodontal Diseases

### 3.1. Expression Levels of the miR-200 Family

With regard to the expression level of the miR-200 family, the data from human studies are still limited. In human gingival keratinocytes, it was indicated that miR-141 is one of the abundantly expressed microRNAs [79]. miR-200a and miR-200c were both found to be decreased in the gingival tissues from patients with periodontitis compared with healthy gingiva [53]. It was found that *Porphyromonas gingivalis* lipopolysaccharide (Pg-LPS) decreased the expression of miR-200c in gingival epithelial cells [35] and gingival tissues in obese mice with periodontitis [54]. However, another study showed that miR-200b was significantly elevated in the gingiva of obese periodontitis subjects using qRT-PCR [55]. miR-200b was also reported to be overexpressed in a Japanese population with chronic periodontitis [80]. Altogether, miR-200a, miR-200b, and miR-200c may exert different functions under inflammatory conditions.

### 3.2. Biological Functions and Clinical Application of the miR-200 Family

Current data suggest that miR-200b and miR-200c may affect the host innate defenses against microbial pathogens. It was shown that mimics of miR-200b and miR-200c (not miR-200a) downregulated NF-κB activation through toll-like receptor (TLR)-4 signaling [81]. They also demonstrated that the LPS-induced expression of the pro-inflammatory molecules, such as interleukin (IL)-6, chemokine (C-X-C motif) ligand (CXCL)-9, and TNF-α in monocytic THP-1 cells, was diminished [81]. Another study showed that miR-200b was induced by the stimulation of IL-1β, TNF-α, and IL-6, and the elevation of miR-200b suppressed the expression of IL-6 and IL-1β in HGFs. Aside from the reciprocal regulation between miR-200b and these inflammatory cytokines, they demonstrated that miR-200b can directly bind to IKKβ and ZEB1 as well [56].

miR-200c was found to inhibit the expression of IL-6, IL-8, and chemokine (C-C motif) ligand (CCL)-5 by directly binding to the 3′ UTRs of these genes and promoting osteogenic differentiation of human bone marrow mesenchymal stromal cells [82]. Similarly, it was shown that miR-200c may alleviate periodontitis, as overexpression of miR-200c reduced the LPS-induced proinflammatory mediators in human gingival fibroblasts (HGFs) and local treatment with miR-200c effectively improved the alveolar bone resorption in a rat model of experimental periodontitis [26]. In obese mice with periodontitis, injection of naked plasmid DNA encoding miR-200c into the gingiva also ameliorated periodontal inflammation [54].

## 4. Oral Submucous Fibrosis (OSF)

As one of the oral potentially malignant disorders, OSF is characterized by the accumulation of extracellular matrix (ECM) components in oral mucosae. The excessive deposition of ECM molecules, such as collagen, is attributed to the persistent activation of myofibroblasts. Myofibroblast participates in the wound healing process with its ability to close a wound and secrete ECM components. A key feature of the activated myofibroblasts is the presence of alpha-smooth muscle actin (α-SMA), which can enhance their contractile ability [83]. It was revealed that the expression of α-SMA is elevated in the oral mucosae of OSF patients [58].

Areca nut-chewing was found to be a crucial factor contributing OSF, and myofibroblast transdifferentiation of buccal mucosal fibroblasts (BMFs) by arecoline stimulation (an alkaloid isolated from areca nut) was considered to eventually lead to the development of OSF. Chang et al. revealed that elevated ZEB1 in areca-nut-associated OSF may contribute to the pathogenesis via activating the α-SMA promoter and inducing myofibroblast activation of BMFs [58]. This result is in line with a study showing that ZEB1 was a direct target of miR-200c and the expression of miR-200c was downregulated in OSF specimen and fBMFs [59]. Likewise, the expression of miR-200b was reduced in OSF tissues and patient-derived fBMFs [57]. miR-200b directly targeted ZEB2 and affected the ZEB2-regulated expression of α-SMA and vimentin in fBMFs. In addition, overexpression of miR-200b induced apoptosis of these myofibroblasts [57].

Collectively, these results suggest that miR-200b and miR-200c are downregulated in OSF, leading to upregulation of ZEB1 and ZEB2. Elevation of ZEB1 and ZEB2 increases the α-SMA and vimentin expression of BMFs, resulting in myofibroblast transdifferentiation.

## 5. Gingival Overgrowth

Drug-induced gingival overgrowth/enlargement refers to medication-related gingival hyperplasia or hypertrophy [84]. Various pharmacologic agents are known to elicit this phenomenon, including anticonvulsants, calcium channel blockers, and immunosuppressants. As a potent immunosuppressant, cyclosporin A (CsA) has been used for the prevention of transplant rejection or treatment of rheumatoid arthritis. Around 50% of adult transplant patients may encounter CsA-associated gingival enlargement [85,86]. It was suggested that administration of CsA may induce EMT in gingival epithelium [62], and the upregulation of the EMT inducer Slug was observed in HGFs [64].

In a rat model of CsA-induced gingival overgrowth, the expressions of miR-200a, miR-200b, and miR-200c were all downregulated in gingival tissues [60]. In agreement with this result, the miR-200b transcript in HGFs was downregulated in response to CsA treatment [63]. Moreover, CsA-inhibited miR-200b led to an increase in Slug expression [63], which is consistent with the CsA-induced EMT theory. Another study demonstrated that the CsA-repressed miR-200a attributed to the gingival overgrowth by upregulation of ZEB2 [61]. Current data indicate that the administration of CsA may suppress the expression level of the miR-200 family in the gingiva, which promotes the changes in EMT.

## 6. Oral Mucositis

The miR-200 family numbers, including miR-200a, miR-200b, miR-200c, and miR-141, were all induced during the formation of radiation-induced oral mucositis in an animal model [65]. Tao et al. demonstrated that the expression of miR-200c was increased dramatically in the normal human keratinocytes after irradiation. They showed that miR-200c was involved in the regulation of cell migration, expression of EMT factors, DNA double-strand break repair, and generation of reactive oxygen species (ROS). Moreover, their results demonstrated that miR-200c participated in the production of proinflammatory cytokines through suppression of NF-κB and Smad2 activation [65].

## 7. Conclusions

Taken together, the miR-200 family seems to be downregulated in the tissues of OSCC, periodontitis, OSF, and gingival overgrowth. They affect the cancer’s aggressiveness and myofibroblast transdifferentiation via directly binding to EMT inducers ZEB1 and ZEB2. The members of the miR-200 family also regulate other targets, such as BMI1, EGFR, IL-6, and IL-8. These data indicate that miR-200s are crucial in the pathogenesis of various dental diseases, and restoration of miR-200s may be a potential strategy to alleviate the progression of these disorders.

## Figures and Tables

**Table 1:** The expression of the miR-200 family in dentistry.

Disease	miRNA	Expression	Target (s) and Factor (s)	Note	Reference
Oral cancer	miR-141	Downregulated	EGFR CDK-4 BCL-2 MMP-2	Reduction of EGFR by overexpression of miR-141 decreases the cell proliferation, apoptosis, migration, and invasion in OSCC cells.	[33]
	miR-141 miR-200c	Downregulated	ZEB1 ZEB2	Overexpression of miR-141 and -200c reduces EMT in TSCC cells.	[34]
	miR-200b miR-200c miR-205	Downregulated	ZEB1	Downregulation of miR-200b, -200c, and -205 during *P.-gingivalis*-induced EMT process in gingival epithelial cells.	[35]
	miR-200b miR-15b	Downregulated	BMI1	Low levels of miR-200b and -15b in chemotherapy-resistant TSCC cells. Upregulation of BMI1 by suppressing miR-200b and -15b induces EMT and confers chemo-resistance.	[36]
	miR-200b	Downregulated	ZEB2 Kindlin-2	Inhibition of ZEB2 and Kindlin-2 by overexpression of miR-200b represses migration and invasion in OSCC cells.	[37]
	miR-200b	-	lncRNA OIP5-AS1	lncRNA OIP5-AS1 increases the expression of fibronectin by sponging miR-200b-3p, results in the chemo-resistance.	[38,39]
	miR-200b	Downregulated	lncRNA MALAT1	Inhibition of MALAT1 by overexpression of miR-200b-3p may promote OSCC development.	[40,41,42]
	miR-200c miR-200b	Downregulated	-	Low levels of miR-200 and -200-5p are correlated with advanced TNM stage and poor prognosis.	[43,44,45]
	miR-200c	Downregulated	BMI1	Inhibition of BMI1 by overexpression of miR-200c reduces stemness and EMT, and sensitizes HNSCCs to chemotherapy.	[46]
	miR-200 family	Dysregulated	-	The miR-200 family is dysregulated in chronic chewing tobacco/cigarette smoke oral keratinocytes.	[47,48]
	miR-200 family	Downregulated	ZEB1 PRPX1	Downregulation of miR-141 and -200c in OSCC cells with upregulation of ZEB1 and PRRX1.	[49]
	miR-200 family	Dysregulated	QKI	A negative feedback loop of QKI/miR-200 maintains the EMT-inducing signals and cell growth.	[50]
	miR-200 family	-	NOTCH1 JUN ETS1	The miR-139-5p, -429, and -200 families modulate stemness and chemo-resistance in cisplatin-resistant OSCC cells by regulating NOTCH1, JUN, and EST1.	[51]
	miR-200 family	Downregulated	NOTCH ZEB1	ZEB1/miR-200 feedback loop maintains stemness in cancer cells by controlling NOTCH signaling.	[52]
Periodontal diseases	miR-200c family	Upregulated	-	High levels in periodontitis gingiva tissues from patients.	[53]
	miR-200c	Downregulated	-	Pg-LPS decreases miR-200c in both periodontitis gingival epithelial cells and tissues.	[54]
	miR-200b	Upregulated	-	High levels of miR-200b in periodontitis gingiva.	[55]
	miR-200b miR-200c	-	IKKβ ZEB1	miR-200b attenuates pro-inflammatory molecules production through IKKβ and ZEB1 in HGFs.	[26,56]
	miR-200c	-	-	Treatment with miR-200c improves alveolar bone resorption and ameliorates periodontal inflammation in animal models.	[26,54]
Oral submucous fibrosis	miR-200b	Downregulated	ZEB2 α-SMA vimentin	Overexpression of miR-200b inhibits the activity of myofibroblast in fBMFs by elevating ZEB2, α-SMA, and vimentin.	[57]
	miR-200c	Downregulated	ZEB1 α-SMA	Overexpression of miR-200c inhibits the myofibroblastic transdifferentiation in fBMFs by elevating ZEB1 and α-SMA.	[58,59]
Gingival overgrowth	miR-200 family	Downregulated	-	miR-200a, -200b, and -200c are downregulated in gingival tissues with CsA-induced overgrowth.	[60]
	miR-200a	Downregulated	ZEB2	CsA-inhibited miR-200a in HGFs may induce EMT and gingival enlargement by upregulating ZEB2.	[61]
	miR-200b	Downregulated	Slug	CsA-inhibited miR-200b in HGFs may induce EMT and overgrowth of HGFs by up-regulating Slug.	[62,63,64]
Oral Mucositis	miR-200 family	Upregulated	-	miR-141, -200a, -200b, and -200c are upregulated during the formation of RIOM in an animal model.	[65]
	miR-200c	Upregulated	-	miR-200c is dramatically increased in irradiated-NHK cells. miR-200c involves in cell proliferation, migration, EMT process, DNA repair, ROS production, and inflammation in irradiated NHK cells.	[65]

## Data Availability

Not applicable.
